# Antioxidative, antifungal, cytotoxic and antineurodegenerative activity of selected *Trametes* species from Serbia

**DOI:** 10.1371/journal.pone.0203064

**Published:** 2018-08-31

**Authors:** Aleksandar Knežević, Mirjana Stajić, Ivana Sofrenić, Tatjana Stanojković, Ivan Milovanović, Vele Tešević, Jelena Vukojević

**Affiliations:** 1 University of Belgrade Faculty of Biology, Takovska, Belgrade, Serbia; 2 University of Belgrade, Faculty of Chemistry, Studentski trg, Belgrade, Serbia; 3 Institute of Oncology and Radiology of Serbia, Pasterova, Belgrade, Serbia; Tallinn University of Technology, ESTONIA

## Abstract

In a last few decades mushrooms are increasingly attracting attention as functional food and sources of biologically active compounds. Several *Trametes* species have been used for centuries in traditional medicine of East Asia cultures, but only *T*. *versicolor* was studied sufficiently while there are less substantial data about medicinal properties of other species. *Trametes versicolor*, *T*. *hirsuta* and *T*. *gibbosa* were the species tested for biological activities. Antifungal potentials of extracts were assessed for clinical strains of selected *Candida* and *Aspergillus* species. ABTS and FRAP assays were used to evaluate antioxidant capacities of studied extracts. Cytotoxic activity was determined against human cervix and lung adenocarcinoma and colon carcinoma cell lines. Antineurodegenerative activity was assessed by determining the rate of acetylcholinesterase and tyrosinase activity. The presence of metabolites in extracts of mycelia and basidiocarps of studied *Trametes* species was analyzed by ^1^H NMR spectroscopy. Studied extracts showed low antifungal potential in comparison with ketoconazole. Basidiocarp extracts were more effective ABTS^+^ scavengers and Fe^2+^ reducers than mycelium ones but less effective in comparison with L-ascorbic acid. Results showed that mycelium extracts had stronger cytotoxic effects against three cancer cell lines than basidiocarp ones, and that cervix adenocarcinoma cells were the most sensitive to the extracts and commercial cytostatics. *T*. *versicolor* mycelium extract was the most effective inhibitor of acetylcholinesterase activity but double weaker than galantamine, and *T*. *gibbosa* mycelium extract was significantly better inhibitor of tyrosinase activity than kojic acid for 40.9%. Chemical analysis indicated strong synergistic action of triterpenes, sugars and polyphenols in applied assays. The results suggest that tested *Trametes* species have significant medicinal potentials which could be attributed to antioxidative and cytotoxic activity. Additionally both, basidiocarps and mycelia extracts can strongly inhibit activity of acetylcholinesterase and tyrosinase.

## Introduction

Mushrooms have an established history of use as healthy food or for preparations in traditional medicine of both the Western and Eastern cultures, but their great pharmacological potential is still underutilized [1]. Among nearly 60 described *Trametes* species just a few are screened for their medicinal properties since records confirmed their use in traditional Chinese medicinal practice for removing toxins, treatment of various infections, strengthening, energy increasing, improvement of liver and spleen function and enhancing of the immune response [2]. *Trametes versicolor* (L.:Fr.) Lloyd whose folk names are Turkey Tail (Western cultures), Yun-Zhi (China), or Kawaratake (Japan), is the most commonly used species from the genus whose whole fruiting bodies are used for tonic or tea preparation [2]. In Ming Dynasty edition of the Compendium of Chinese Materia Medica, more than 120 strains of *T*. *versicolor* have been recorded for their medicinal properties which were the best manifested after chronic use [2]. Medicinal effects have been also demonstrated in conventional medicine mainly in treatment of various types of cancers, infections of the respiratory, urinary and digestive tracts, chronic hepatitis and rheumatoid arthritis [2, 3]. Some reports also implied traditional usage of polypore mushroom fruiting bodies for increasing memory and improvement of mental functions [4]. Extensive and controlled *in vitro* researches also reported antiviral and antioxidant activity of polysaccharopeptides isolated from *Trametes* spp. fruiting body extracts [5, 6].

All these activities are based on the production of numerous active compounds, such as two proteoglucans (PSK and PSP), phenolic compounds, terpenoids and organic acids. PSK and PSP are extracted from *T*. *versicolor* basidiocarps and primarily have imunostimulatory activities, i.e. induce production of interleukin-6, interferons, imunoglobuline-G, macrophages and T-lymphocytes. However, they also posses immunosuppressive potential in chemotherapy, radiation and blood transfusion, antiproliferative effect on various tumour cell lines based on stimulation of superoxide dismutase and gluthation peroxidase productions, as well as antimicrobial, antiviral, analgetic and hepatoprotective activities [7]. Phenolic compounds as well as linoleic acid isolated from *T*. *lactinea* are good antimicrobial, antioxidative, cytostatic and genoprotective agents [6, 8, 9, 10]. Significant antibacterial effect is characteristic of sesquiterpenoid coriolin firstly isolated from *T*. *consors*, while ascorbic acid was shown as the main carrier of antioxidative activity in *T*. *versicolor* [9, 11]. Since described activities might have been responsible for medicinal effects in traditional practice the goal of this study was to elucidate antifungal, antioxidative, cytotoxic and antineurodegenerative potential of basidiocarp and mycelium extracts of selected *Trametes* species.

## Materials and methods

### Organisms and cultivation conditions

The basidiocarps were collected from Serbia, and identified according to the macroscopic features and the micromorphology of the reproductive structures [12, 13] as *Trametes gibbosa*, *T*. *hirsuta* and *T*. *versicolor*. Small fragment of each fresh fruiting body was extracted on Malt agar medium (MA) for isolation of pure cultures of *T*. *gibbosa* BEOFB 310, *T*. *hirsuta* BEOFB 301 and *T*. *versicolor* BEOFB 321, which are then maintained in the Culture Collection of the Institute of Botany, Faculty of Biology, University of Belgrade (BEOFB) (Table 1). For the locations of sampling no specific permission was required according to Serbian Law on Nature Protection (“Official Gazette of RS”, no. 36/2009, 88/2010 and 91/2010 –corr. and 14/2016). Studied species are not endangered or protected, according to IUCN list or any other domestic and international legislative.

**Table 1:** Geographic origin of studied *Trametes* species.

Species	Code of strain	Origin of strain	Names of the locations	Geographic coordinates
*Trametes gibbosa* (Pers.) Fr.	BEOFB310	Suva Mt., Serbia	Devojački grob	N 43°19ˈ89.27˝, E 22°14ˈ25.68˝
*Trametes hirsuta* (Wulf.:Fr.) Pil.	BEOFB301	Suva Mt., Serbia	Kraljev put	N 43°20ˈ41.77˝, E 22°13ˈ76.76˝
*Trametes versicolor* (L.:Fr.) Lloyd	BEOFB321	Suva Mt., Serbia	Kraljev put	N 43°20ˈ63.89˝, E 22°13ˈ53.58˝

Inoculum preparation was consisted of several phases: *(i)* inoculation of 100.0 mL synthetic medium (glucose– 10.0 g/L; NH_4_NO3−2.0 g/L; K_2_HPO4−1.0 g/L; NaH_2_PO_4_ × H_2_O – 0.4 g/L; MgSO_4_ × 7H_2_O – 0.5 g/L; yeast extract– 2.0 g/L; pH 6.5) with 25 mycelial plugs (Ø 0.5 cm, from 7-day-old culture on malt agar medium); *(ii)* incubation at room temperature (22 ± 2 °C) on a rotary shaker (100 rpm) for 7 days; *(iii)* washing of obtained biomass with sterile distilled water (dH_2_O) 3 times; *(iv)* homogenisation of biomass with 100.0 mL sterile dH_2_O in laboratory blender.

Mycelial biomasses of the studied species were obtained by submerged cultivation in 1000-mL flasks containing 500.0 mL of modified synthetic medium (with glucose in the concentration of 65.0 g/L) inoculated with 30.0 mL of homogenized inoculum, at room temperature and on a rotary shaker during 21 days.

### Extraction

Dried mycelia and fruiting bodies (3.0 g) were ground to powder in laboratory blender. Extraction was carried out in 96% ethanol (90.0 mL) on magnetic stirrer during 72 hours. The obtained extracts were centrifuged (20 °C, 3000 rpm, 10 min), and supernatants filtrated through Whatman No. 4 filter paper. The filtrates were concentrated under reduced pressure in a rotary evaporator (Büchi, Rotavapor R-114, Switzerland) at 40 °C to dryness, and redissolved in 96% ethanol (for antioxidative assay), or in 5% dimethyl sulphoxide (DMSO) (for antifungal and anticancer assays) or in 0.1 M Na-phosphate buffer (for antineurodegenerative potential assay) to an initial concentration of 32.0 mg/mL. The extraction yield was determined as the ratio of dry fungal biomass before extraction and dry extract weight after the evaporation process.

### Determination of antifungal activity

Antifungal potentials of basidiocarp and mycelium extracts of selected *Trametes* species were determined for *Candida albicans* (BEOFB 811m), *C*. *krusei* (BEOFB 821m), *C*. *parapsilosis* (BEOFB 831m), *Aspergillus glaucus* (BEOFB 301m), *A*. *flavus* (BEOFB 221m) and *A*. *fumigatus* (BEOFB 232m). Cultures of the micromycetes were provided by the Institute of Public Health of Serbia (Belgrade, Serbia) and maintained on malt agar medium at 4 °C in the culture collection of the Institute of Botany, Faculty of Biology, University of Belgrade.

The tested micromycetes were cultivated on Sabouraud dextrose agar (SDA) at temperature of 37 °C for 21 days. Cell/spore suspensions were prepared by washing agar surface with sterile 0.9% saline enriched with 0.1% Tween-a 80 (v/v). The obtained suspensions were squeezed through double sterile gauze and mixed at vortex and thereafter cell/spore number was defined by hemocytometer. Level of turbidity was determined spectrophotometrically at 530 nm and cell/spore number was adjusted to 10^6^ CFU/mL with sterile saline [14].

DMSO extracts of fruiting bodies and mycelia were sterilized by filtration through Whatman No. 4 filter paper and 0.2 μm membrane filter. Antifungal potentials of extracts were determined by microdilution method, using 96-well microtiter plates [15]. Series of double extract dilutions (from 32.0 to 0.5 mg/mL) were analyzed. Each well contained Sabouraud dextrose broth (SDB), cell/spore suspension and extract of a defined concentration. Oxido-reduction indicator of micromycete growth, resazurin (10.0 μL), was added to the mixture after 24 hours of incubation at 37 °C and cultivation was continued for next 48 hours. Mixture without extract was used as a negative control, while a positive control contained commercial antimycotic, ketoconazole, instead extract. The tested ketoconazole concentrations were ranged from 0.031 to 0.002 mg/mL (series of double dilutions). The effect of 5% DMSO on cell growth and spore germination was also analyzed by its addition to the mixture instead SDB. The lowest extract concentration without visible mycelium growth was defined as the minimal inhibitory concentration (MIC), and that without growth after reinoculation of 2.0 μL of the mixture on SDA as the minimal fungicidal concentration (MFC) [16].

### Determination of antioxidative activity

Antioxidative activities of basidiocarp and mycelium extracts of the studied species were determined by two methods, ABTS and FRAP tests.

**ABTS test** is based on measuring the level of ABTS stock solution colour change [17].The initial solution of ABTS cation radicals was prepared by dissolution of 9.0 μg ABTS in 2.5 mL dH_2_O and addition of 44.0 μL 140 mM potassium persulphate (K_2_S_2_O_8_) solution, 12 to 16 hours before experiment beginning, while the stock solution was prepared immediately prior to measurement by dilution of the initial solution with dH_2_O and adjustment of solution absorbance on 0.700 ± 0.020 at 734 nm. The reaction mixture (1500.0 μL of the ABTS stock solution and 15.0 μL of extract of concentration of 1.0 mg/mL) was incubated at room temperature for 4 min and absorbance change was measured spectrophotometrically at 734 nm. Distilled water was used as a blank. Extract concentration required for ABTS^+^ reduction, equivalent to reduction of 1.0 mg/mL ascorbic acid (AAEC), was determined using equation of calibration curve for ascorbic acid. EC_50_ value (mg extract/mL) presents effective extract concentration that scavenging 50% ABTS cation radicals and it is obtained by linear regression analysis.

**FRAP test** presents measuring the level of change of ferric tripyridyl triazine (Fe(III)(TPTZ)_2_) complex colour during reduction of ferrous form (Fe (II)-TPTZ) which has an intense blue colour [18]. The solution of FRAP reagent (300 mM acetic buffer (pH 3.6), 10 mM solution of 2,4,6-Tri(2-pyridyl)-s-triazine(TPTZ) in 40 mM HCl, 20 mM FeCl_3_ × 6H_2_O) was prepared immediately prior to measurement and heated at 37 °C. Absorbance of the reaction mixture (3000.0 μL of FRAP reagent and 100.0 μL of extract) was measured spectrophotometrically at 593 nm after 4 min of incubation at 37 °C. The mixture which contained dH_2_O instead extract was used as a negative control and FRAP reagent as a blank. Antioxidative activity was defined using equation of calibration curve for FeSO_4_ × 7H_2_O according to the formula:

F(mM)=(Σ/changeofstandardabsorbance)×FRAPvaluesfor1mMofstandard

F–equivalent of reduced Fe^2+^, Σ –change of sample absorbance, standard–L-ascorbic acid. The results were presented as equivalent of reduced Fe^2+^expressed in mM.

### Determination of cytotoxic activity

Human cervix adenocarcinoma (HeLa), human colon carcinoma (LS174) and human lung adenocarcinoma (A549) cell lines, obtained from the American Type Culture Collection (ATCC), were used in the study. These cancer cell lines were maintained in the recommended Roswell Park Memorial Institute (RPMI) 1640 medium supplemented with 100.0 g/L heat-inactivated (56 °C) fetal bovine serum (FBS), 3.0 mM L-glutamine, 100.0 mg/mL streptomycin, 100 IU/mL penicillin, and 25.0 mM 4-(2 hydroxyethyl)-1-piperazineethanesulfonic acid (HEPES) and pH 7.2 adjusted with bicarbonate solution. Cells were grown in a humidified atmosphere of 95% air/5% CO_2_ (v/v) at 37 °C.

Stock solution of extracts (100.0 mg/mL), prepared in 50.0 g/LDMSO, was dissolved in enriched RPMI 1640 medium to the required working concentrations. Inoculation of 100.0 μL of medium per microtiter plate well was performed with 2000 neoplastic HeLa, 7000 LS174 or 5000 A549 cells. MRC5 cell line of human fetal fibroblast (5000 cells per wall) was used for assessment of the potential of selective action of tested extracts on cancer cell lines. RPMI 1640 medium was used as a blank. After 24 hours of incubation and cell adhesion, 50.0 μL of extract, at the concentrations of 200.0, 100.0, 50.0, 25.0 and 12.5 μg/mL, was added per well, while the same amount of fresh medium was added in the blanks and wells with control cells. Cell cultures were incubated at 37 °C for 72 hours.

Effects of extracts on survival of cell lines were determined by microculture tetrazolium test (MTT test), i.e. by measuring of tetrazolium salt reduction to insoluble purple coloured formazan in living cells [19, 20]. After 72-hour-old incubation, 20.0 μL of MTT solution [3-(4,5-dimethylthiazol-2-yl)-2,5-diphenyltetrazolium bromide in phosphate-buffering saline] at the concentration of 5.0 mg/mL was added to each wall. The samples were incubated at 37 °C in humidified atmosphere of 95% air/5% CO_2_ (v/v) for 4 hours and thereafter 100.0 μL of 10% sodium dodecylsulphate (SDS) dissolved in 0.01 M HCl was added in order to dissolve the insoluble formazan. After 24 hours, cell growth inhibition rate was determined by ELISA microplate reader at 575 nm and calculated according to the formula:

$$Cell\;growth\;inhibition\;rate\;\left(\%\right)=(A_{c}-A_{s})\;/A_{c}\times 100$$

A_c_−absorbance of the control, A_s_−absorbance of sample.

IC_50_value was defined as the concentration of the extract inhibiting cell survival by 50% compared with a negative control (DMSO instead extract). Commercial cytostatics, *cis*-diamminedichloroplatinum (*cis*-DDP) and doxorubicin were used as a positive control.

### Antineurodegenerative activity assays

#### Determination of acetylcholinesterase inhibitory activity

Acetylcholinesterase (AChE) activity inhibition rate was determined spectrophotometrically using 96-well microtiter plates by method of Ellman et al. [21]. The reaction mixture (140.0 μL of 0.1 mM sodium phosphate buffer (pH 8.0), 20.0 μL of 5,5′-dithiobis(2-nitrobenzoic acid) (DTNB), 20.0 μL of extract, 20.0 μL of AChE) was incubated at 25 °C for 15 min. Reaction was initiated by adding of 10.0 μL of acetylthiocholine iodide which hydrolysis was accompanied by a change of absorbance at 412 nm due to transformation of DTNB to yellow 5-thio-2-nitrobenzoate anion (TNB^2-^) in the reaction catalyzed by AChE, 15 min after initiation. Mixture of 5% DMSO and sodium phosphate buffer (pH 8.0) was used as a blank. AChE activity inhibition rate was determined according to the formula:

AChEactivityinhibitionrate(%)=[(E−S)/E]×100

E–enzyme activity without extract, S–enzyme activity with extract.

The obtained values were compared with commercial inhibitor of AChE, galantamine.

#### Determination of tyrosinase inhibitory activity

Tyrosinase activity inhibition rate was determined spectrophotometrically using 96-well microtiter plates by method of Likhitwitayawuid and Sritularak [22]. The reaction mixture (80.0 μL of 66.7 mM phosphate buffer (pH 6.8), 40.0 μL of extract, 40.0 μL of tyrosinase (46 U/L) dissolved in phosphate buffer) was incubated at 23 °C for 10 min and thereafter 40.0 μL of 2.5 mM L-DOPA was added. Absorbance was measured after 30 min of incubation at 475 nm. Tyrosinase activity inhibition rate was determined according to the formula:

Tyrosinaseactivityinhibitionrate(%)=[((A−B)−(C−D))/(A−B)]×100

A–absorbance of tyrosinase in phosphate buffer, B–absorbance of phosphate buffer, C–absorbance of the reaction mixture, D–absorbance of extract in phosphate buffer.

### Determination of phenol and flavonoid contents

Contents of soluble phenolic compounds in the extracts of fruiting bodies and mycelia of the studied species were determined using Folin-Ciocalteu reagent and gallic acid as standards [23]. The reaction mixture (mixture of 1000.0 μL of 10% Folin-Ciocalteu reagent and 200.0 μL of sample of concentration of 1.0 mg/mL was incubated for 6 min in dark and thereafter enriched with 800.0 μL of Na_2_CO_3_) was incubated on rotary shaker (100 rpm) in dark at room temperature for 2 hours. Absorbance was measured spectrophotometrically at 740 nm. The mixture without extract was used as a blank. Total phenol content was presented as gallic acid equivalent (GAE) per mg of dried extract using equation of calibration curve for GAE.

Total flavonoid contents in extracts were determined by method of Park et al. [24]. The reaction mixture (1000.0 μL of extract of concentration of 1.0 mg/mL, 4100.0 μL of 80% ethanol, 100.0 μL of 10% Al(NO_3_)_3_ × 9H_2_O and 100.0 μL of 1.0 M water solution of potassium acetate) was incubated in dark on a rotary shaker (100 rpm) and room temperature for 40 min and thereafter absorbance was measured at 415 nm. The mixture which contained ethanol instead extract was used as a blank. Total flavonoid content was presented as μg of quercetin equivalent (QE) per mg of dried extract using equation of calibration curve for QE.

### ^1^H NMR spectroscopy

All NMR spectra were measured on Bruker (Billerica, MA, USA) AVANCE III spectrometer (500.26 MHz for ^1^H nuclei), using 5 mm broad-band probehead. Samples were dissolved in standard buffer solution 90 mM KH_2_PO_4_ in D_2_O (pH 6.0) and spectra were obtained at 298 K.

### Statistical analysis

The assays were carried out in five replicates and results are expressed as mean ± standard error. One-way analysis of variance (ANOVA) and Tukey`s HSD post-hoc test were performed to test any significant differences among means. Statistical significance was declared at P < 0.01. All statistical analyses were done using software STATISTICA, version 6.0 (StatSoft, Inc., Tulsa, USA).

## Results

### Extraction yield

Yields of extractions of fruiting bodies and mycelia of *Trametes gibbosa* BEOFB 310, *T*. *hirsuta* BEOFB 301 and *T*. *versicolor* BEOFB 321 in 96% ethanol differed statistically as among species as between somatic and reproductive phases (P<0.01). Yield of mycelium extraction was higher than basidiocarp one and it was ranged from 8.00 ± 0.27% in *T*. *versicolor* to 34.60 ± 0.27% in *T*. *gibbosa*. Contrary to the results for mycelium, the highest level of fruiting body extraction was noted in *T*. *versicolor* (6.67 ± 0.07%) and the lowest in *T*. *gibbosa* (2.20 ± 0.02%) (Table 2).

**Table 2:** Yields (%) of selected *Trametes* spp. basidiocarp and mycelium extractions.

Species	Basidiocarps	Mycelium
*Trametes gibbosa*	2.20 ± 0.02 C^a^	34.60 ± 0.27 A
*Trametes hirsuta*	2.85 ± 0.08 B	12.00 ± 0.19 B
*Trametes versicolor*	6.67 ± 0.07 A	8.00 ± 0.12 C

^a^ Means with different letters within a column are significantly different(P<0.01)

### Antifungal activity

Levels of sensitivity of 6 human pathogens to ethanolic extracts of basidiocarps and mycelia of the tested species of the genus *Trametes* were different (Table 3). Mycelial extracts showed higher antifungal potential in comparison with basidiocarp ones. Thus *T*. *hirsuta* mycelium extract inhibited the growth of *Candida krusei* at concentration of only 4.0 mg/mL and at the concentration of 32.0 mg/mL growth of other tested species. This extract at concentration of 32.0 mg/mL had also fungicidal effect on *Aspergillus glaucus*. Basidiocarps extracts inhibited growth of a small number of micromycetes and *T*. *hirsuta* extract had the highest capacity among tested species because it inhibited *C*. *parapsilosis* growth at concentration of 16.0 mg/mL. Based on MIC and MFC values analyzed extracts showed significantly weaker antifungal potential in comparison with commercial antimycotic ketoconazole which MIC was ranged between 0.0078 mg/mL (*Candida albicans*, *C*. *krusei*, *C*. *parapsilosis*, *Aspergillus flavus*, *A*. *fumigatus*) and 0.0156 mg/mL (*A*. *glaucus*) and MFC from 0.0078 mg/mL (*A*. *flavus*) to 0.0313 mg/mL (*A*. *glaucus*). Antifungal potential of tested *Trametes* species was decreased by the next order: *T*. *hirsuta* > *T*. *versicolor* > *T*. *gibbosa*.

**Table 3:** Antifungal activities of ethanolic extracts of selected *Trametes* spp. basidiocarps (B) and mycelia (M) and commercial antimycotic.

Species	*Trametes gibbosa*				*Trametes hirsuta*				*Trametes versicolor*				Ketoconazole	
	MIC (mg mL^-1^)		MFC (mg mL^-1^)		MIC (mg mL^-1^)		MFC (mg mL^-1^)		MIC (mg mL^-1^)		MFC (mg mL^-1^)		MIC (mg mL^-1^)	MFC (mg mL^-1^)
	B	M	B	M	B	M	B	M	B	M	B	M		
*Candida albicans* (BEOFB 811m)	nd	32.0	nd	nd	32.0	32.0	nd	nd	nd	32.0	nd	nd	0.0078	0.0156
*Candida krusei* (BEOFB 821m)	nd	nd	nd	nd	nd	4.0	nd	nd	nd	8.0	nd	nd	0.0078	0.0156
*Candida parapsilosis* (BEOFB 831m)	32.0	32.0	nd	nd	16.0	32.0	nd	nd	nd	nd	nd	nd	0.0078	0.0156
*Aspergillus glaucus* (BEOFB 301m)	nd	32.0	nd	nd	nd	32.0	nd	32.0	nd	32.0	nd	nd	0.0156	0.0313
*Aspergillus flavus* (BEOFB 221m)	nd	32.0	nd	nd	nd	nd	nd	nd	nd	nd	nd	nd	0.0078	0.0078
*Aspergillus fumigatus* (BEOFB 232m)	nd	32.0	nd	nd	nd	32.0	nd	nd	nd	nd	nd	nd	0.0078	0.0156

nd–not detected

Species of the genus *Candida* were more sensitive to tested extracts then species of the genus *Aspergillus* and sensitivity was decreased by the order: *C*. *parapsilosis > C*. *albicans > A*. *glaucus > C*. *krusei > A*. *fumigates > A*. *flavus*. However, mycelial extracts of *T*. *gibbosa* (at subinhibitory concentrations) and *T*. *hirsuta* (at concentrations higher than 2.0 mg/mL) caused conidium demelanisation in the most resistant *A*. *flavus*.

### Antioxidative activity

Extracts of fruiting bodies and mycelia of selected *Tramates* species showed significant antioxidative potentials which varied depending on species and material used for extraction. Basidiocarp extracts had significantly higher capacity than mycelium ones (P<0.01), but lower in comparison with commercial antioxidant, L-ascorbic acid (Table 4).

**Table 4:** Antioxidative activities of ethanolic extracts of selected *Trametes* spp. basidiocarps and mycelia and commercial antioxidant.

Species	Extract	EC_50_ [mg mL^-1^]	Equivalent of reduced Fe^2+^ [mM]
		ABTS	FRAP
*Trametes gibbosa*	basidiocarps	14.67 ± 0.93 E^a^	0.015 ± 0.001 D
	mycelium	31.43 ± 0.83 A	0.011 ± 0.001 E
*Trametes hirsuta*	basidiocarps	18.33 ± 1.41 D	0.013 ± 0.003 D
	mycelium	27.50 ± 0.42 B	0.016 ± 0.004 D
*Trametes versicolor*	basidiocarps	8.46 ± 0.49 F	0.037 ± 0.007 B
	mycelium	20.00 ± 1.57 C	0.025 ± 0.00 C
L-ascorbic acid		0.247 ± 0.012 G	2.282 ± 0.023 A

^a^ Means with different letters within a column are significantly different (P<0.01)

Extracts of *T*. *versicolor* fruiting bodies and mycelium had the highest ability of ABTS radical scavenging and Fe^2+^reduction. EC_50_ of basidiocarp and mycelium extracts in the radical neutralization were 8.46 ± 0.49 mg/mL and 20.00 ± 1.57 mg/mL, respectively, while amounts of reduced Fe^2+^ were 0.037 ± 0.007 and 0.025 ± 0.000 mM, respectively. Extracts of *T*. *gibbosa* mycelium had the lowest antioxidative capacity, i.e. EC_50_ value in ABTS^+^ scavenging was 31.43 ± 0.83 mg/mL and amount of reduced Fe^2+^ was only 0.011 ± 0.001 mM (Table 4).

Studies of phenol and flavonoid contents in the extracts of selected *Trametes* species showed that they were present both in fruiting bodies and mycelia (Table 5). Among the tested species, fruiting bodies and mycelium of *T*. *gibbosa* were the richest with phenols (18.21 ± 0.30 and 14.79 ± 0.57 μg GAE/mg, respectively) and flavonoids (2.85 ± 0.06 and 3.46 ± 0.08 μg QE/mg, respectively) contrary to *T*. *hirsuta* extracts where amounts of phenol were less in even 50.85% and 16.09%, respectively, and flavonoid in 35.09% and 31.79%. Since very low correlation between contents of the compounds and radical neutralization levels was found (R^2^ values for fruiting body phenols and flavonoids were 0.19 and 0.15, respectively, and for mycelium ones 0.01 and 0.00, respectively), it was concluded that they were not the main or the only carriers of antioxidative potentials of the species.

**Table 5:** Phenol and flavonoid contents in ethanolic extracts of selected *Trametes* spp. basidiocarps and mycelia.

Species	Extract	Phenol content (μg GAE mg^-1^ of dried extract)	Flavonoid content (μg QE mg^-1^ of dried extract)
*Trametes gibbosa*	basidiocarps	18.21 ± 0.30 A^a^	2.85 ± 0.06 C
	mycelium	14.79 ± 0.57 B	3.49 ± 0.08 A
*Trametes hirsuta*	basidiocarps	8.95 ± 0.30 D	1.85 ± 0.00 E
	mycelium	12.41 ± 0.24 C	2.36 ± 0.03 D
*Trametes versicolor*	basidiocarps	14.18 ± 0.30 B	1.51 ± 0.03 F
	mycelium	14.51 ± 0.27 B	3.21 ± 0.11 B

^a^ Means with different letters within a column are significantly different (P<0.01)

### Cytotoxic activity

Extracts of studied *Trametes* species showed lower cytotoxic effects on tested cancer cell lines in comparison with commercial cytostatics, *cis*-DDP and doxorubicin, which were used as a positive control (Table 6). Mycelium extracts had stronger cytotoxic effects than basidiocarp ones. Comparing studied species, it was shown that *T*. *hirsuta* was a species with the highest cytotoxic potential contrary to *T*. *gibbosa* which basidiocarp and mycelium extracts were the weakest anticancer agents. Screening of IC_50_ values showed that they were ranged from 21.01 ± 1.95 μg/mL for *T*. *hirsuta* mycelium extract against HeLa cells to over 200.00 μg/mL for the same extract against other tested cell lines and for *T*. *versicolor* basidiocarp extract against LS174, A549 and MRC5, as well as that HeLa cells were the most sensitive to tested extracts and commercial cytostatics (Table 6).

**Table 6:** Cytotoxic activities of selected *Trametes* spp. basidiocarp and mycelium extracts and commercial cytostatics.

Species	Extract	IC_50_ [μg mL^-1^]			
		HeLa	LS174	A549	MRC5
*Trametes gibbosa*	basidiocarps	193.53 ± 8.91 B^a^	>200.00 A	>200.00 A	>200.00 A
	mycelium	>200.00 A	>200.00 A	>200.00 A	>200.00 A
*Trametes hirsuta*	basidiocarps	116.13 ± 3.14 C	158.46 ± 37.15 B	177.66 ± 20.17 B	175.70 ± 1.45 B
	mycelium	21.01 ± 1.95 E	45.75 ± 0.02 D	57.94 ± 5.08 C	145.17 ± 6.48 C
*Trametes versicolor*	basidiocarps	168.54 ± 28.90 B	>200.00 A	>200.00 A	>200.00 A
	mycelium	42.40 ± 0.74 D	86.12 ± 12.34 C	65.57 ± 7.75 C	77.37 ± 0.82 D
*cis-*DDP*		2.10 ± 0.20 F	5.54 ± 1.03 E	11.92 ± 2.19 D	14.44 ± 1.90 E
Doxorubicin*		0.62 ± 0.30 G	3.78 ± 0.49 F	0.24 ± 0.02 E	0.46 ± 0.18 F

^a^ Means with different letters within a column are significantly different (P<0.01)

*Commercial cytostatic

### Potential of acetylcholinesterase and tyrosinase inhibition

**Fig 1:**
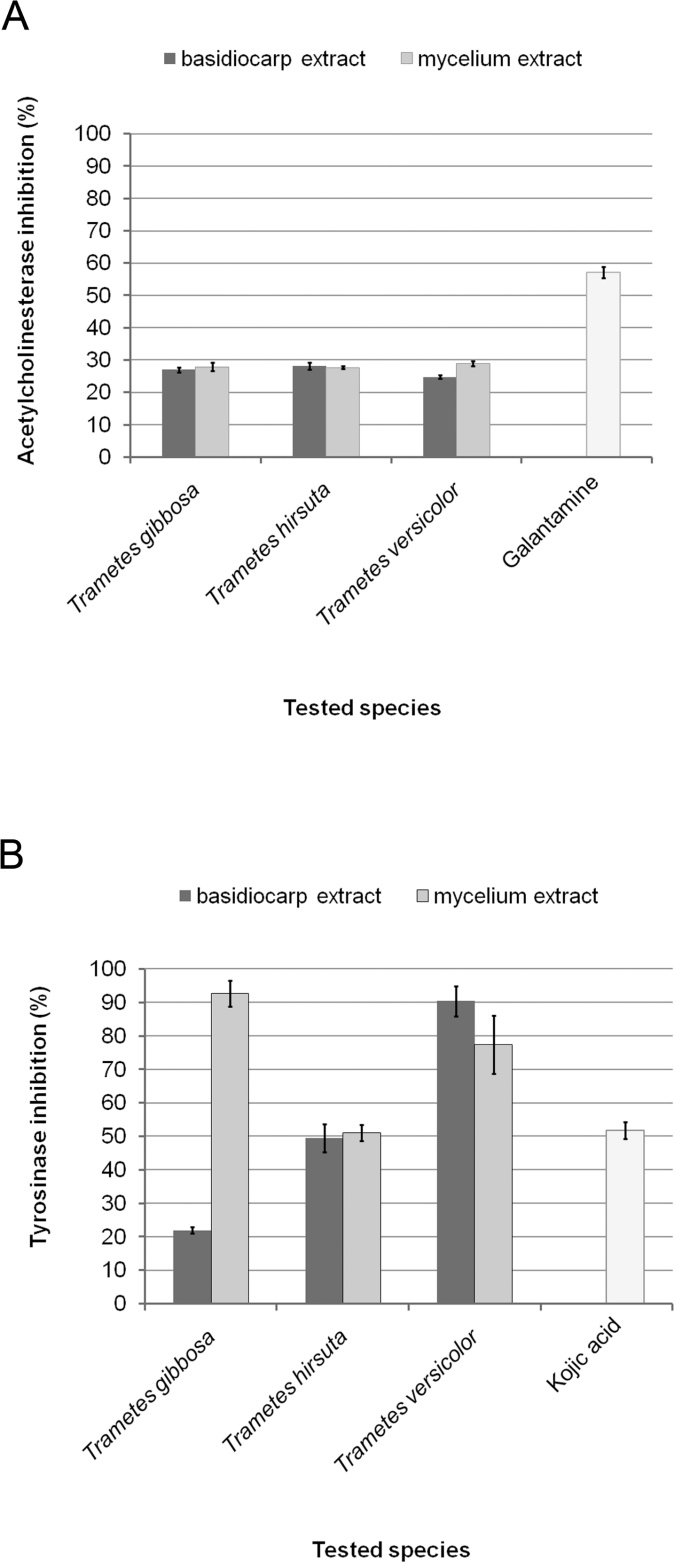
Potential of basidiocarp and mycelium extracts of selected *Trametes* species and commercial inhibitors to inhibit activities of acetylcholinesterase (A) and tyrosinase (B) at concentration of 100.0 μg/mL.

Basidiocarp and mycelium extracts of the studied species showed significant potentials of inhibition of acetylcholinesterase and tyrosinase activities in comparison with synthetic inhibitors, galantamine and kojic acid, respectively (Fig 1A and 1B). Extents of AChE activity inhibition were ranged between 24.7 ± 0.6% and 28.9 ± 0.8%, and statistically significant differences between basidiocarp and mycelium extracts were not noted except in the case of *T*. *versicolor* where mycelium extract was significantly better inhibitor (P<0.05). *T*. *versicolor* mycelium extract was the most effective, namely it inhibited AChE activity in 28.9 ± 0.8% which was almost double less than commercial inhibitor galantamine (57.1 ± 1.7%) (Fig 1A).

In the case of tyrosinase, high extents of activity inhibition (ranged from 49.5 ± 4.2% to 92.7 ± 3.9%) were noted for majority of extracts, except for *T*. *gibbosa* basidiocarp one where inhibition was significantly lower, only 21.9 ± 1.0% (Fig 1B). *T*. *gibbosa* was also unique species according to statistically significant difference between basidiocarp and mycelium extracts in levels of tyrosinase activity inhibition (P<0.001), which was not observed in other two studied species. In comparison with kojic acid as a synthetic inhibitor of tyrosinase activity which inhibited 51.8 ± 2.5% of the enzyme at concentration of 100.0 μg/mL, *T*. *versicolor* extracts were significantly better inhibitors of the enzyme (Fig 1B).

### ^1^H NMR spectroscopy

**Fig 2:**
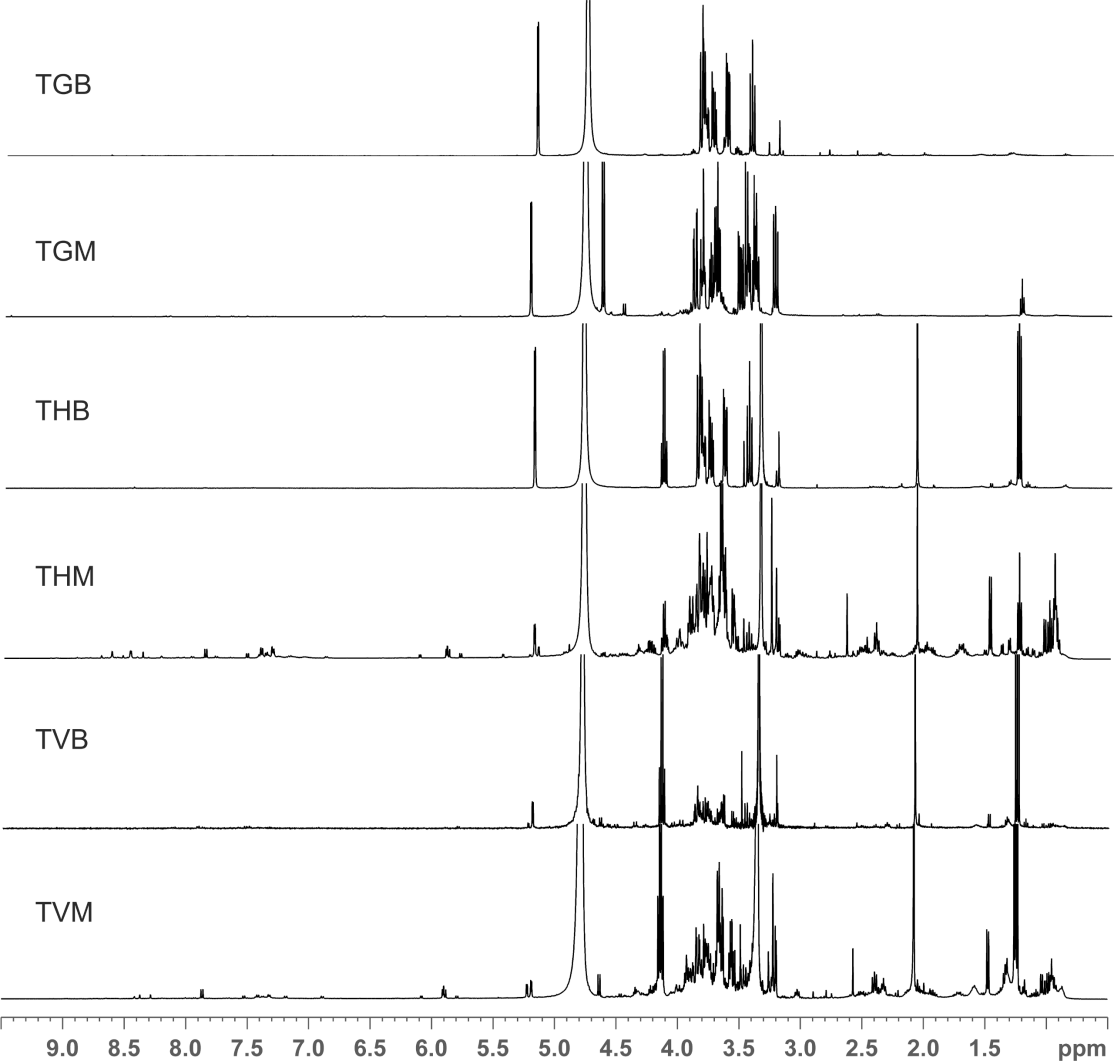
The full ^1^H NMR spectra of ethanol extracts of selected *Trametes* species. *T*. *gibbosa* basidiocarp (TGB), *T*. *gibbosa* mycelium (TGM), *T*. *hirsuta* basidiocarp (THB), *T*. *hirsuta* mycelium (THM), *T*. *versicolor* basidiocarp (TVB), *T*. *versicolor* mycelium (TVM).

**Fig 3:**
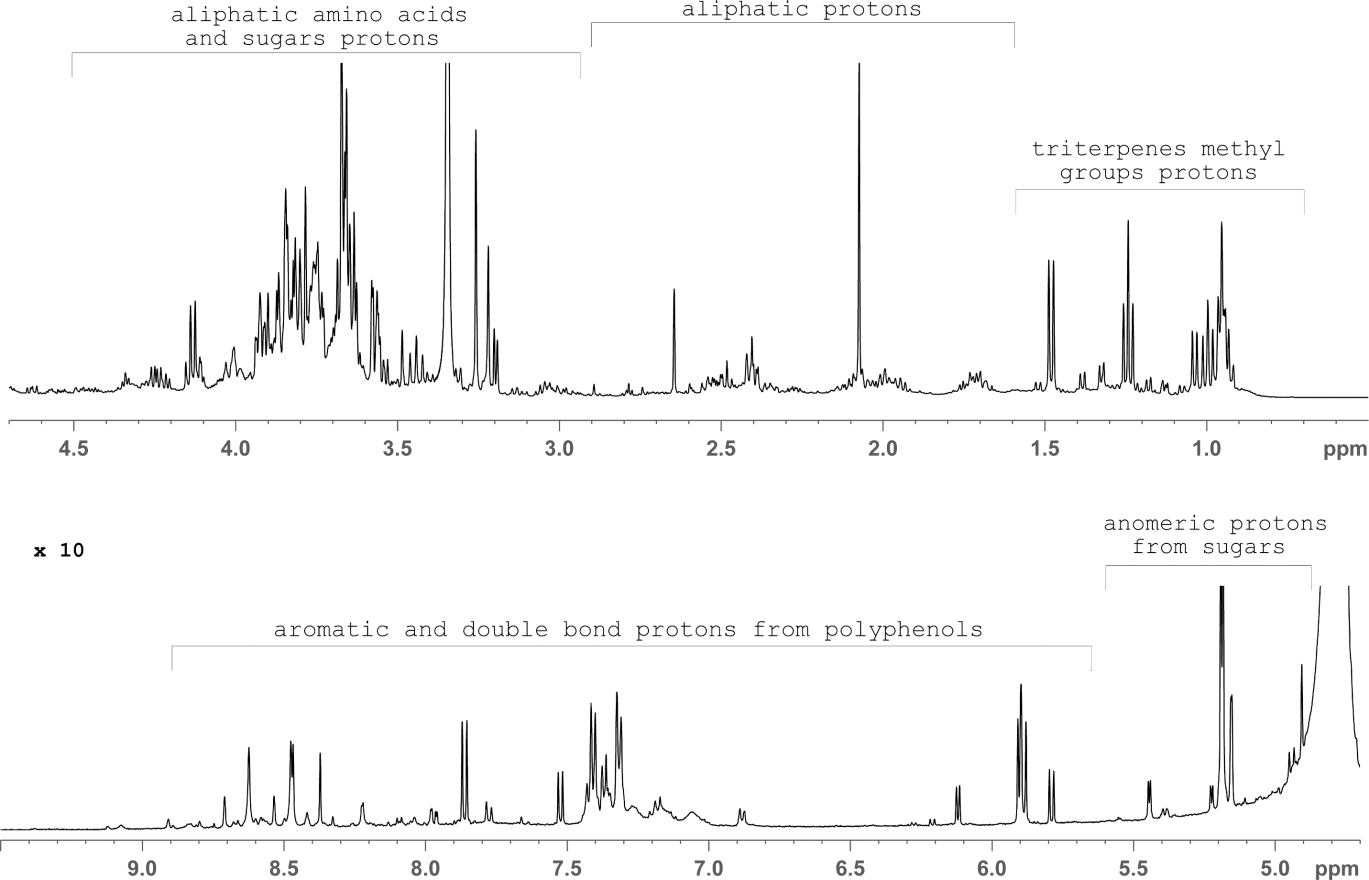
^1^H NMR spectrum of *Trametes hirsuta* mycelium extract with labeled peaks from appropriate group of compounds.

In the present study, the ethanol extracts of mycelia and basidiocarps of studied *Trametes* species were analyzed by ^1^H NMR spectroscopy. The obtained NMR spectra of extracts of all examined species showed the presence of metabolic variations between mycelia and basidiocarps (Fig 2). The major differences in NMR spectra were in the region δ6.5–8.5ppm (S1 Fig). This chemical shifts reported for different polyphenols. Differentiations in a region of δ0.8–1.3 ppm, were from methyl groups of triterpenes. Sugars were present in all extracts i.e., signals around 5 ppm are from anomer protons of sugars and in the area between δ3.0–4.5 ppm are signals from sugars and aliphatic amino acids (Fig 2). Results indicated that mycelium extract of *T*. *hirsuta* was the richest with metabolites as there were signals of triterpenes, sugars and polyphenols (Fig 3). Correlating to this, ethanol extract of *T*. *hirsuta* mycelium shows the best activities in all tests conducted, except in tyrosinase inhibition. On the other hand, in basidiocarp extract of *T*. *hirsuta* the main components were mostly sugars and they have very low activities. The NMR profile of *T*. *versicolor* mycelium is similar to *T*. *hirsuta* and has similar test results. *T*. *gibbosa* has the poorer ^1^H NMR spectra profile for both mycelium and basidiocarp extracts. Dominant signals came from sugars (δ3.0–4.5 ppm). This extract shows less activity in all tests, and NMR profiles are compatible with that result.

## Discussion

The previous results showed that biomass extractability depends on species, strain and solvent [25, 26, 27]. Thus Ren et al. [26] demonstrated that yield of *Trametes gibbosa* basidiocarp extraction in petroleum ether, ethyl acetate and methanol was rather different. Methanol was also good solvent for *T*. *versicolor* fruiting body extraction and extraction yields were ranged between 4.10% and 9.16% [25, 27]. Based on numerous results it can be concluded that yield of extraction is higher in alcohols than in other solvents, as well as that methanol is better solvent than 96% ethanol.

Numerous studies showed that various basidiocarp and mycelium extracts of the *Trametes* species possess antifungal properties [28, 29, 30]. Using disc-diffusion method, Yamaç and Bilgili [28] reported that acetone extract of *T*. *versicolor* fruiting bodies was highly active against *Saccharomyces cerevisiae* but without any effect on *C*. *albicans*. Contrary to acetone extract, ethyl acetate and dichloromethane extracts of these species basidiocarps at the same concentration had no antifungal effect. Later, using microdilution method, Sivaprakasam et al. [29] and Hleba et al. [30] showed that *T*. *versicolor* fruiting body extract inhibited the growth of *C*. *albicans* (MIC was higher than 1.0 mg/mL), that *A*. *flavus*, *A*. *fumigatus* and *A*. *niger* were slightly sensitive to methanolic and aqueous extracts of *T*. *hirsuta* basidiocarps, and that methanolic extracts had higher antifungal potential compared with aqueous but still significantly weaker in comparison with commercial antimycotic.

Although previous studies showed that phenolic compounds presented in fruiting bodies and mycelia of the *Trametes* species are the main carriers of antifungal activity, terpenoids, polysaccharides PSK and PSP and cinnamic acid also have this role [7, 10, 11]. Antifungal activities of polyphenols depend on their chemical structure and polarity, i.e. lack of polar groups increases the lipophilicity and facilitates diffusion through the cell membrane [31]. According to Daglia [32] this activity of phenol compounds is based on presence of highly reactive hydroxyl groups as well as parts which have high affinity for binding for proteins that primarily inhibit telomerase and lipoxygenase and interactions with signalling transduction pathways of membrane receptors. However, Cowan [33] emphasized that phenol antifungal activity can also be realized by inhibition of oxidative phosphorylation though sulfhydryl groups as well as various nonspecific interactions.

Morpho-physiological changes, including demelanization of reproductive structures, in the species of the genus *Aspergillus* exposed to the effects of individual plant extract compounds as well as macromycete extracts were observed by De Billerbeck et al. [34] and Souza et al. [35]. Mossier et al. [36] pointed out that conidia depigmentation can be attributed to the effects of phenolic compounds present in extracts of numerous macromycetes which inhibit melanin synthesis. Melanin is able to absorb 50–75% UV-light and neutralize free radicals generated by action of host macrophags and neutrophils that contribute to spore resistance and survival as well as to virulence of pathogen micromycetes, while demelanization can significantly decrease their extent of pathogenicity [37, 38]. This property can be important, especially in the case of the *Aspergillus* species, because some of them cause serious mycosis and mycotoxicosis in human and animals [39].

Species of the genus *Trametes* are also characterized by significant antioxidative potential [6, 40, 41]. Kamiyama et al. [41] reported that, depending on solvent, extract of *T*. *versicolor* fruiting bodies can neutralise up to 50% DPPH•, and Johnsy and Kaviyarasana [40] that methanolic extract of *T*. *gibbosa* basidiocarps reduced even 91.5% of these radicals. Ethanolic extracts of *T*. *gibbosa* BEOFB 310, *T*. *hirsuta* BEOFB 301 and *T*. *versicolor* BEOFB 321 had slightly lower capacity of DPPH radical scavenging [42] but higher in comparison with ethanolic extracts of *T*. *hirsuta* strain studied by Sheikh et al. [6]. Additionally, it was shown for several mushroom species to possess moderate or even high capacity to neutralise ABTS radicals or to have ferric ion reducing power [43, 44]. ABTS radical scavenging for methanolic extract was ranged from 20% to 90% and for *T*. *consors* was approximately 47% [43]. Results presented in the study of Gan et al. [44] demonstrated that ethanolic and aqueous extracts of *Agaricus bisporus* and *A*. *brasiliensis* could reduce ferric ions producing the Fe^2+^ equivalents in the amounts ranged from 0.084 to 0.187 mM, which was higher reducing power comparing to the studied *Trametes* species. It was previously shown that phenols are essential for free radical scavenging in fungi due to the presence of hydroxyl groups which have role of neutralizers, metal chelators and hydrogen donors [45]. These compounds are found in fruiting bodies and mycelia of nearly all macromycetes and their amounts vary from species to species with significantly higher content in basidiocarps [46]. However, comparing the results obtained for DPPH• scavenging by *T*. *gibbosa* BEOFB 310 [42] and strain studied by Johnsy and Kaviyarasana [40] it can be concluded that phenols are not the only carriers of antioxidative activity. Namely, content of phenols in *T*. *gibbosa* BEOFB 310 basidiocarp extract which scavenged 63.5% of DPPH radicals was only 15.7% lower than in the extract of another strain which reduced even 91.5% of the radicals. In the case of flavonoids, concentration in *T*. *gibbosa* BEOFB 310 was about 92% higher than in another strain and this significant difference can be attributed to either various polarity of solvents or different capacity of their synthesis.

Although the results demonstrated that in most cases cytotoxic effects of extracts were weaker on control cell line than on cancer cells, only *T*. *hirsuta* mycelium extract showed satisfactory level of selectivity to tested cancer cell lines.

Numerous searches showed that extracts as well as compounds isolated from various species of the genus *Trametes* possess significant cytotoxic potential [8, 26, 47, 48]. Abilities of ethanolic extracts of *T*. *hirsuta* and *T*. *versicolor* fruiting bodies and mycelia to suppress proliferation of HeLa, LS174 and A549 cell lines *in vitro* are in accordance with the results obtained by Lau et al. [47] and Janjušević et al. [49] for ethanolic extract of *T*. *versicolor* basidiocarps which IC_50_ values for promyeloid leukaemia (HL-60 and NB-4), Burkitt lymphoma (Raji), human breast adenocarcinoma (MCF-7) and liver hepatocellular carcinoma (Hep2G) cell lines were ranged between 123.5 and 269.3 μg/mL. In comparison with the results of these authors, cytotoxic activity of ethanolic extract of *T*. *versicolor* BEOFB 321 mycelium was significantly higher, considering that IC_50_ values for all studied cell lines were less than 100.0 μg/mL. Likewise, based on the results of these authors as well as Harhaji et al. [8] for mouse B16 melanoma cell line it can be assumed that proliferation inhibition and apoptosis induction are mechanisms of cytotoxic activities of extracts. Hsieh et al. [50] showed that antiproliferative effects of *T*. *versicolor* ethanolic extracts are based on arrest of cell cycle in G_0_/G_1_ phase and Harhaji et al. [8] reported that fruiting body methanolic extract of this species arrests cycle of melanoma B16 cells in S and G_2_/M phase. Cytotoxic activity of extracts of the studied species can be attributed to proteoglucans, terpenoids and phenol compounds which are soluble in ethanol and that are found in basidiocarp and mycelium extracts of *Trametes* species [8, 26, 51]. Considering that compounds of *Trametes* spp. extracts showed significant positive results in clinical trials in patients suffering from different cancers [3, 7] and that cytotoxic effect is the feature of majority of antitumor drags [8], it can be said that potentials of studied species, especially selectivity of *T*. *hirsuta* mycelium extract in comparison with the control cell line, are from great importance for future researches of possible use in the cancer treatment.

Selected species of the genus *Trametes* were for the first time tested in terms of inhibition of AChE and tyrosinase activities, which was preliminary reported by Knežević et al. [52]. Previous searches showed that extracts of few fungal species can contain compounds which inhibit AChE activity [53, 54]. This activity in mushrooms is attributed to terpenoids and alkaloids which inactivate AChE by binding for active centre or peripheral binding sites [53, 55]. In comparison with AChE activity inhibition rate, which was obtained by El-Hadi et al. [56] for *Emericella unguis* extracts (80%, at concentration of 200.0 μg/mL), inhibition level of extracts of selected *Trametes* species, at concentration of 100.0 μg/mL, can be considered significant. Extracts of these species were shown as significantly stronger inhibitors of tyrosinase activity than *Aspergillus sydowii* extracts (36%, at concentration of 200.00 μg/mL) and commercial inhibitor, kojic acid [57]. According to Şenol et al. [58], this higher potential of tested extracts to inhibit activities of AChE and tyrosinase in comparison with individual compounds can be attributed to a synergistic interaction of numerous compounds of the crude extracts.

## Conclusion

According to the presented results, extracts of *Trametes gibbosa*, *T*. *hirsuta* and *T*. *versicolor* have significant medicinal potentials based on several groups of biologically active compounds and their synergistic effects. The present study gave a novel approach in complementary strategies for therapies of cancers and neurodegenerative disorders. Further studies should go towards isolation, characterization and testing of individual active compounds of the extracts with the aim of obtaining new drugs and creation of successful therapies.

## Supporting information

S1 Fig ^1^H NMR spectra of ethanol extracts of selected *Trametes* species in area from 5 to 10 ppm. *T*. *gibbosa* basidiocarp (TGB), *T*. *gibbosa* mycelium (TGM), *T*. *hirsuta* basidiocarp (THB), *T*. *hirsuta* mycelium (THM), *T*. *versicolor* basidiocarp (TVB), *T*. *versicolor* mycelium (TVM).
(TIF)
